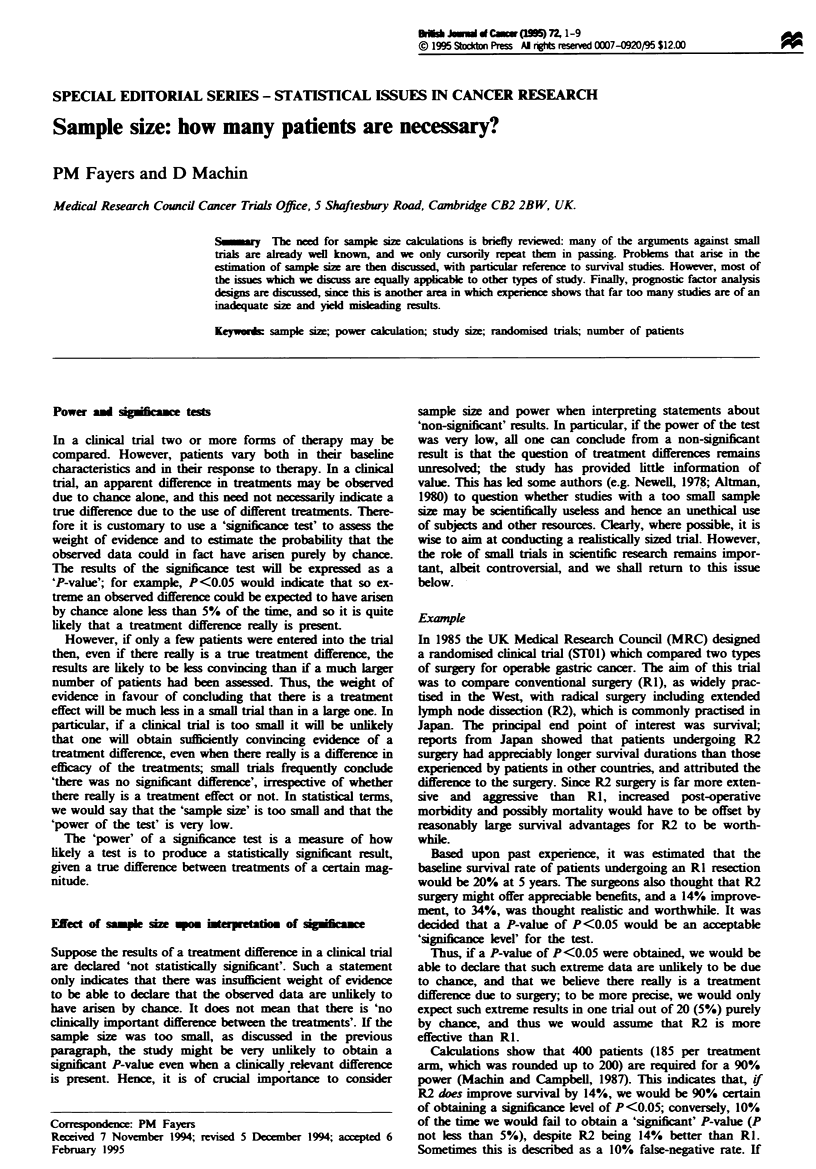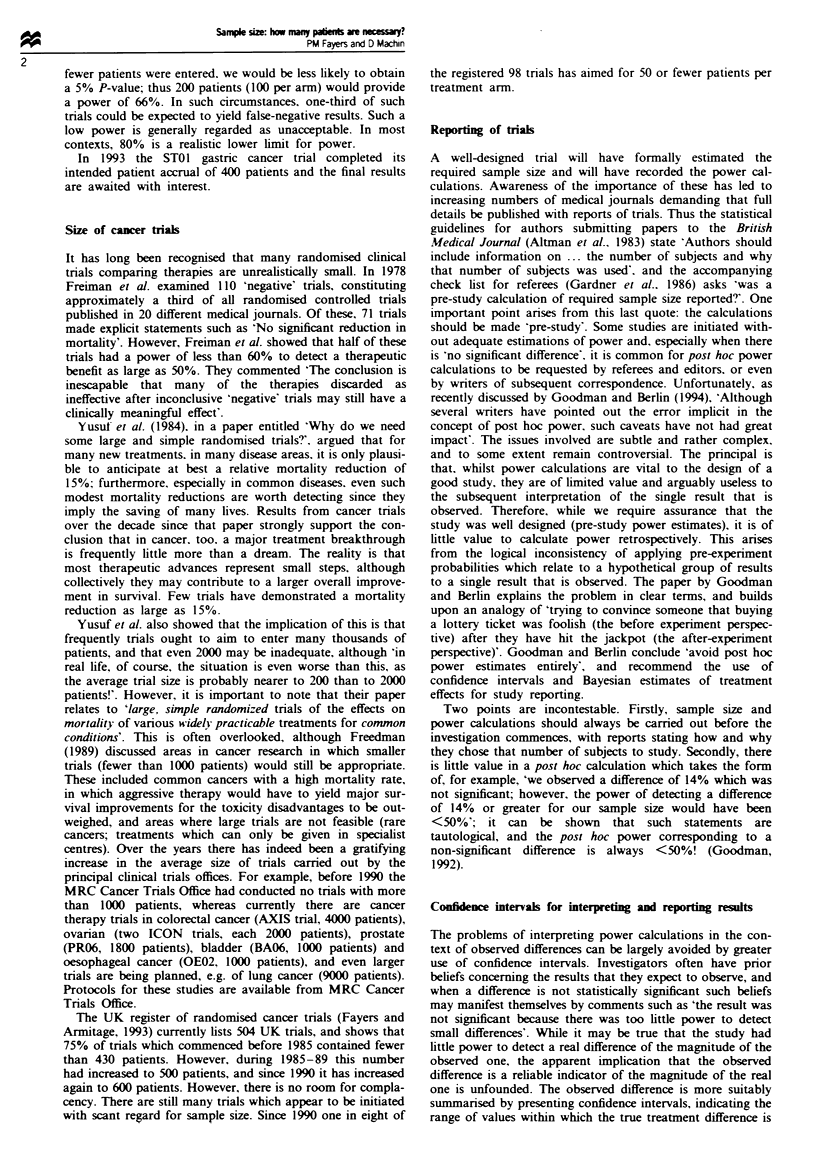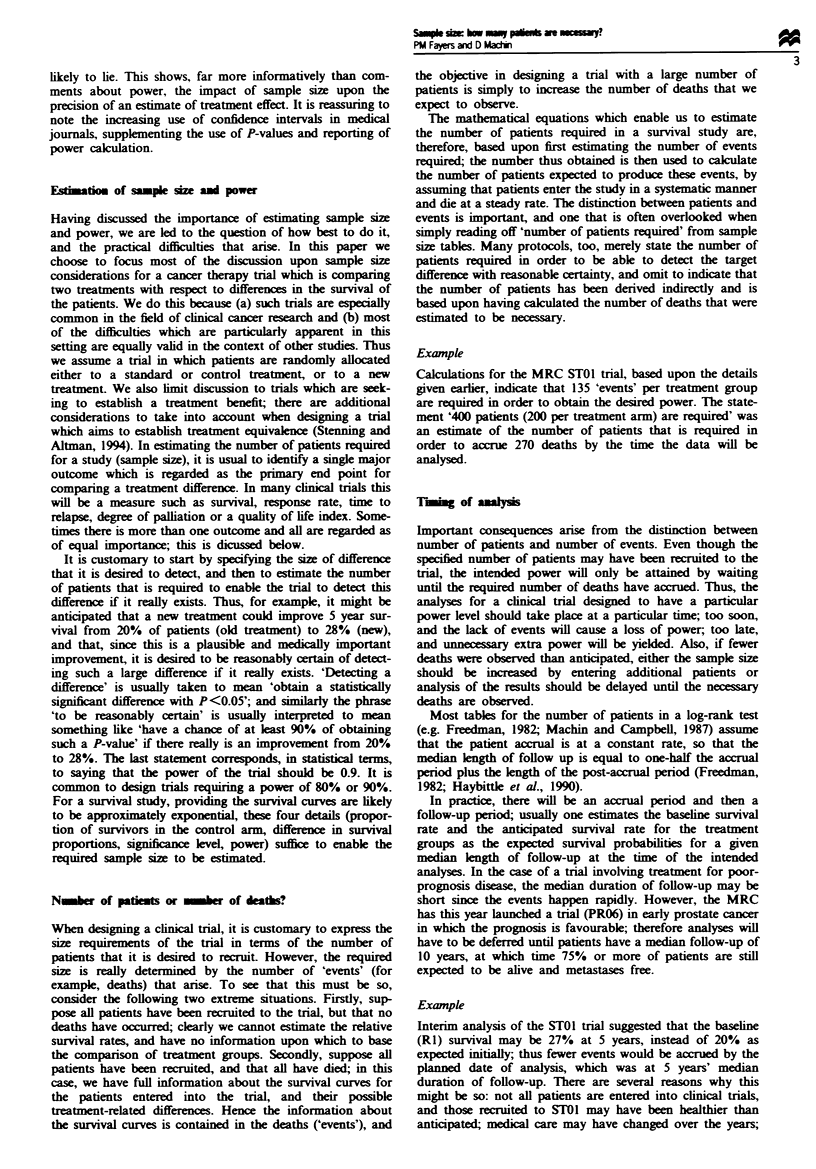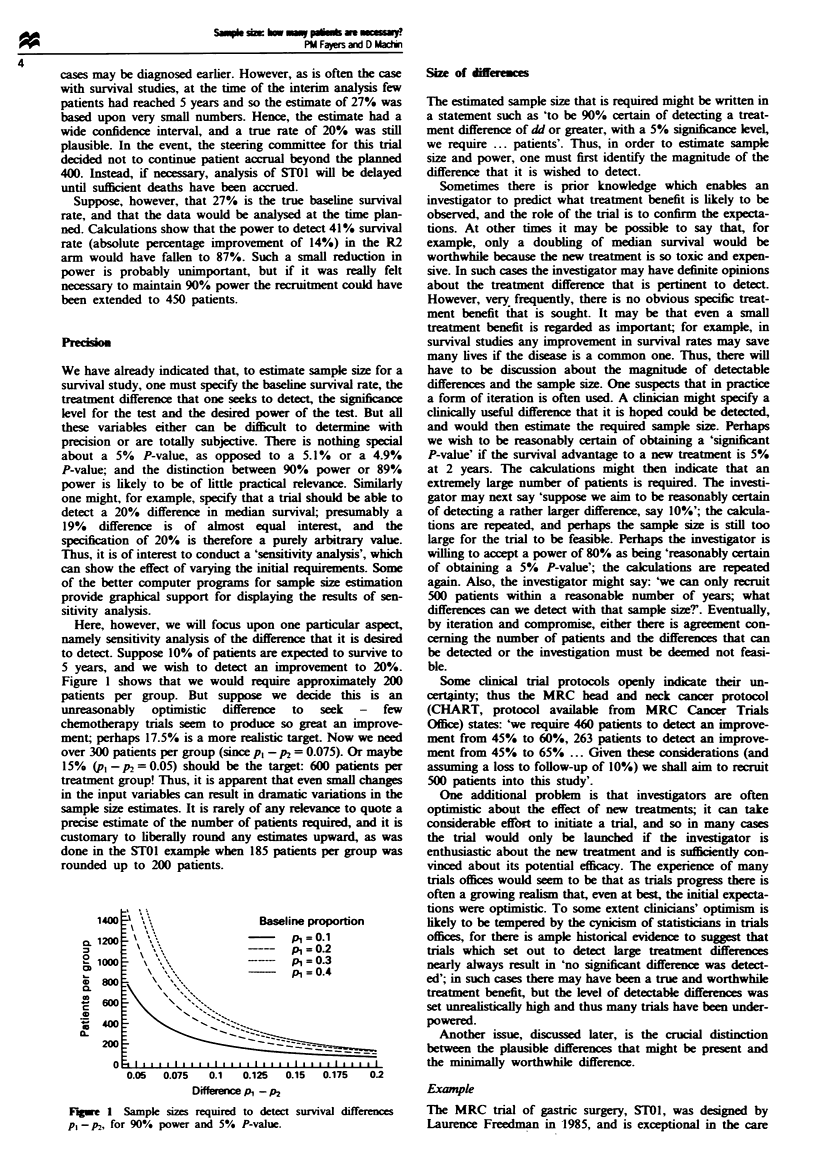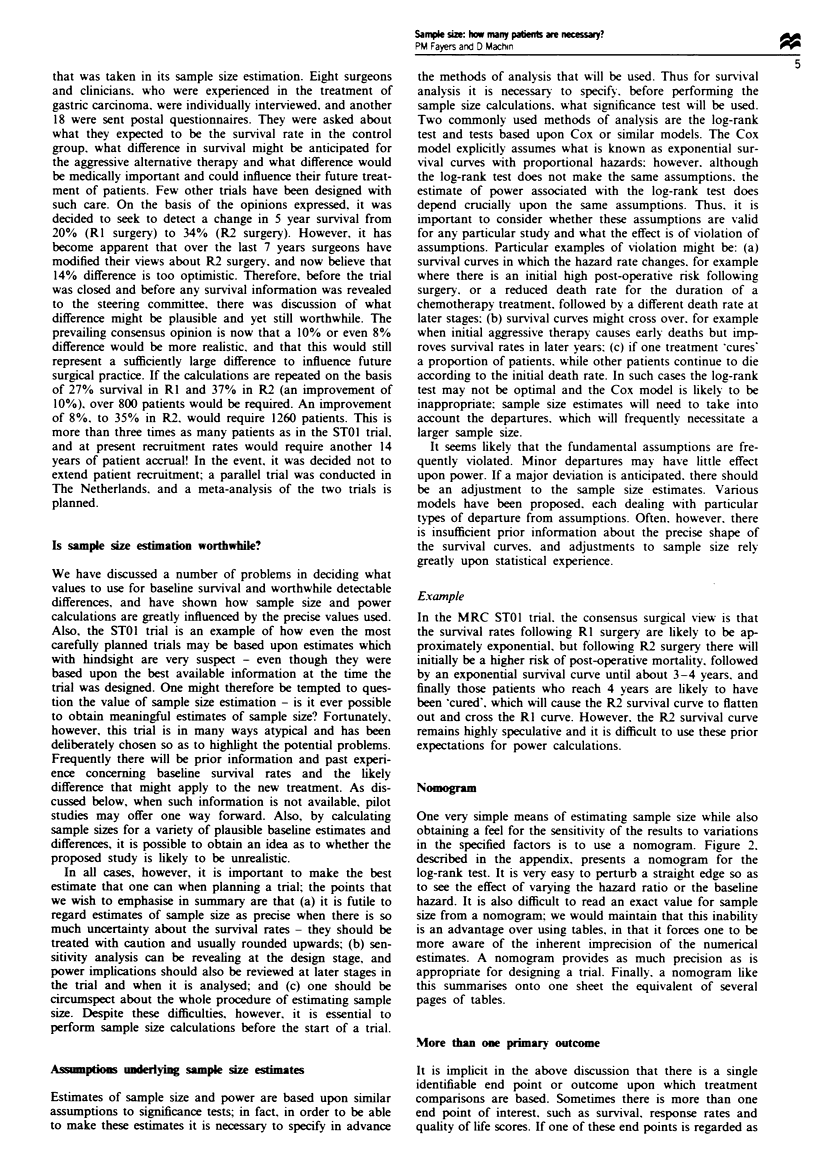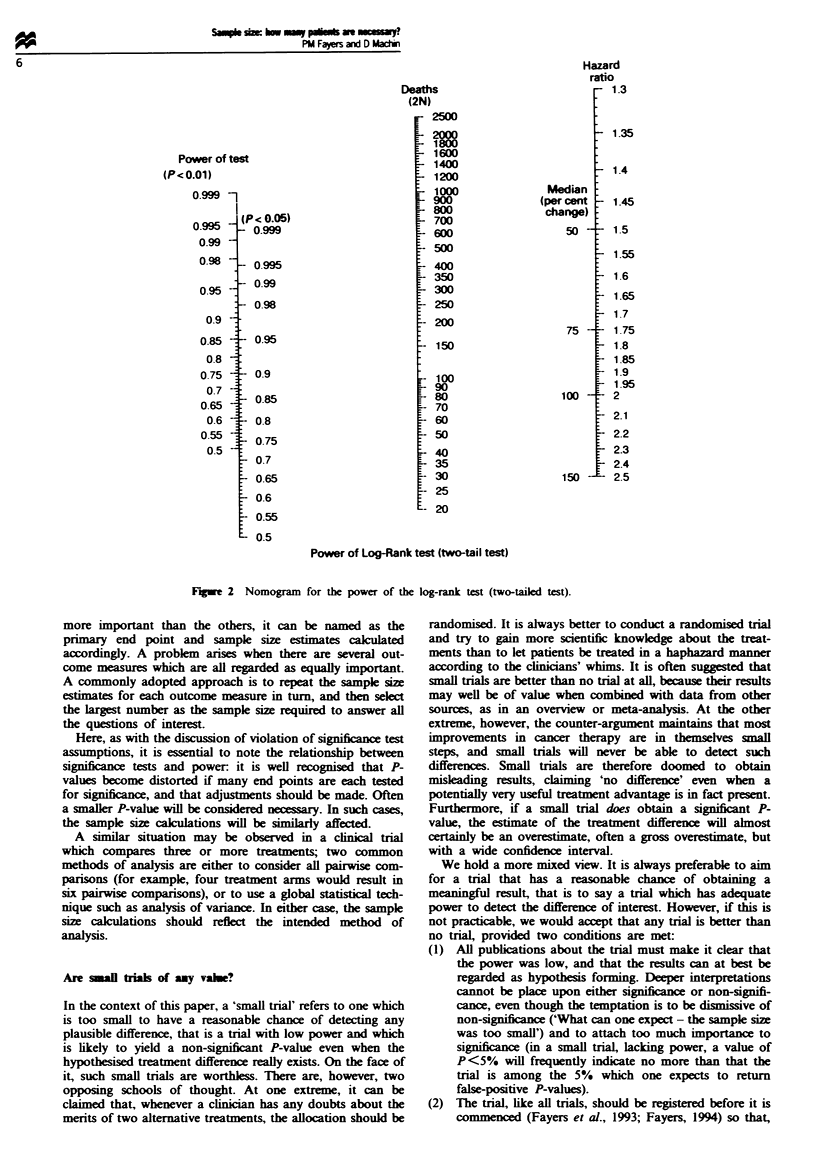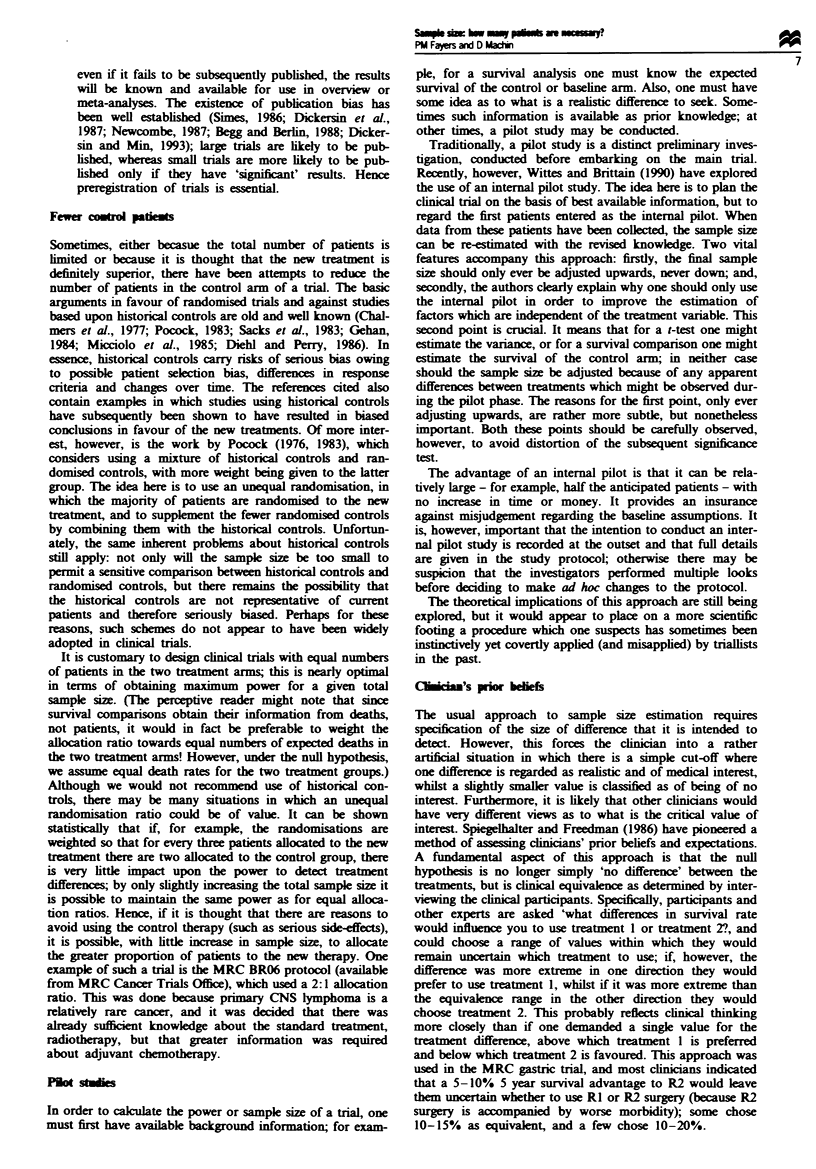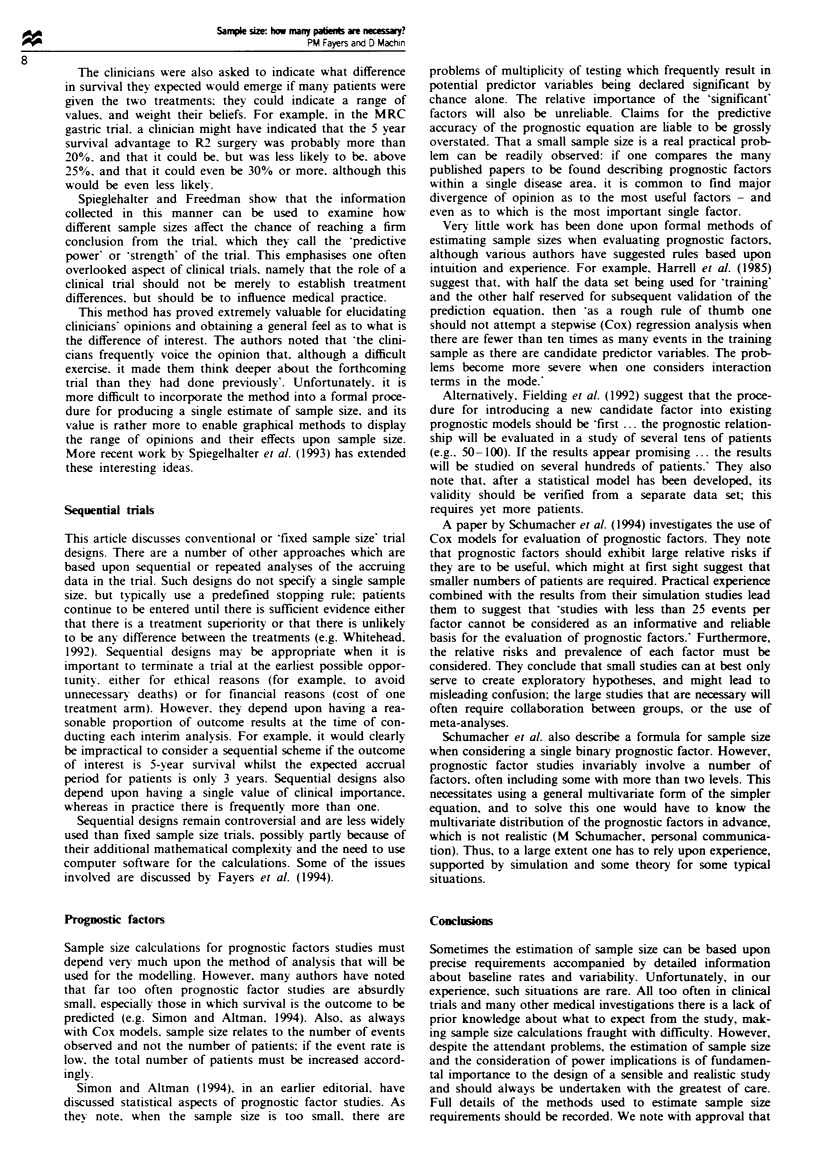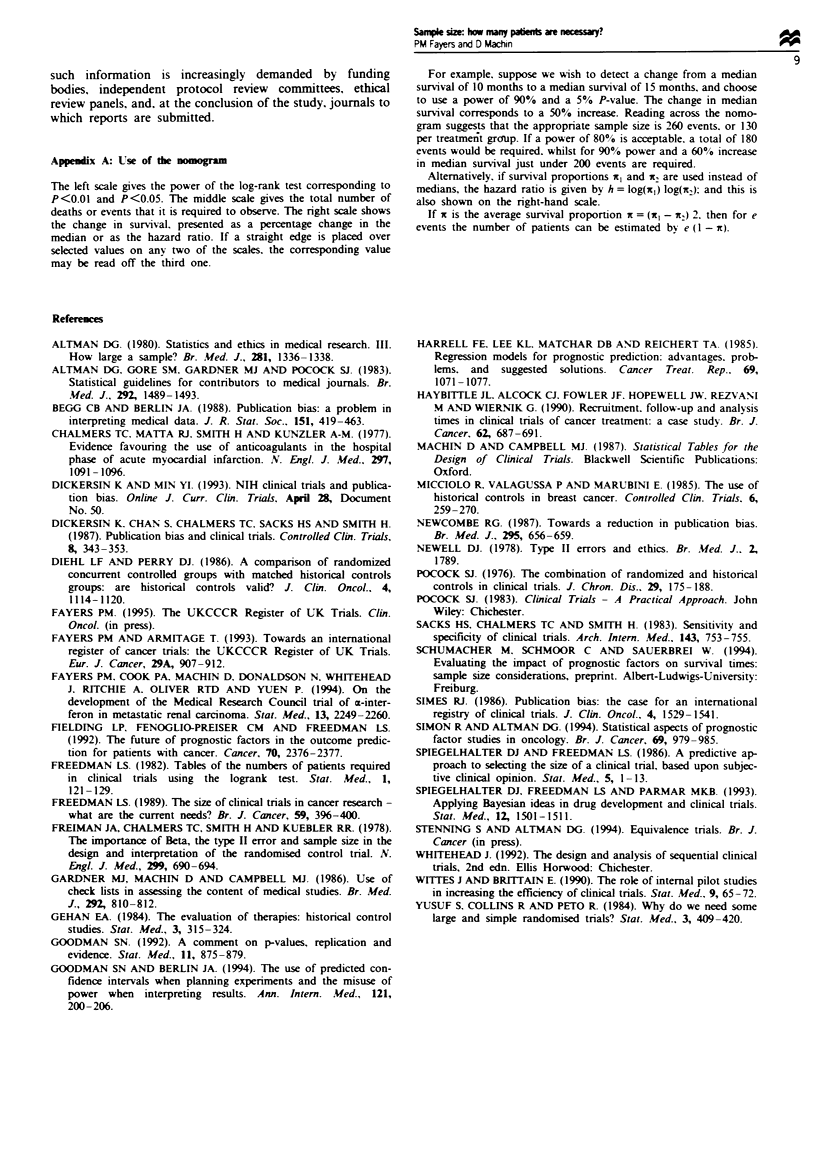# Sample size: how many patients are necessary?

**DOI:** 10.1038/bjc.1995.268

**Published:** 1995-07

**Authors:** P. M. Fayers, D. Machin

## Abstract

The need for sample size calculations is briefly reviewed: many of the arguments against small trials are already well known, and we only cursorily repeat them in passing. Problems that arise in the estimation of sample size are then discussed, with particular reference to survival studies. However, most of the issues which we discuss are equally applicable to other types of study. Finally, prognostic factor analysis designs are discussed, since this is another area in which experience shows that far too many studies are of an inadequate size and yield misleading results.


© 1995 Stockton Press All rights reserved 0007-0920/95 $12.00

SPECIAL EDITORIAL SERIES - STATISTICAL ISSUES IN CANCER RESEARCH

### Sample size: how many patients are necessary?

PM Fayers and D Machin

*Medical Research Council Cancer Trials Office, 5 Shaftesbury Road, Cambridge CB2 2BW, UK.*

**Summary** The need for sample size calculations is briefly reviewed: many of the arguments against small trials are already well known, and we only cursorily repeat them in passing. Problems that arise in the estimation of sample size are then discussed, with particular reference to survival studies. However, most of the issues which we discuss are equally applicable to other types of study. Finally, prognostic factor analysis designs are discussed, since this is another area in which experience shows that far too many studies are of an inadequate size and yield misleading results.

**Keywords:** sample size; power calculation; study size; randomised trials; number of patients

### Power and significance tests

In a clinical trial two or more forms of therapy may be compared. However, patients vary both in their baseline characteristics and in their response to therapy. In a clinical trial, an apparent difference in treatments may be observed due to chance alone, and this need not necessarily indicate a true difference due to the use of different treatments. Therefore it is customary to use a 'significance test' to assess the weight of evidence and to estimate the probability that the observed data could in fact have arisen purely by chance. The results of the significance test will be expressed as a '*P*-value'; for example, $P<0.05$ would indicate that so extreme an observed difference could be expected to have arisen by chance alone less than 5% of the time, and so it is quite likely that a treatment difference really is present.

However, if only a few patients were entered into the trial then, even if there really is a true treatment difference, the results are likely to be less convincing than if a much larger number of patients had been assessed. Thus, the weight of evidence in favour of concluding that there is a treatment effect will be much less in a small trial than in a large one. In particular, if a clinical trial is too small it will be unlikely that one will obtain sufficiently convincing evidence of a treatment difference, even when there really is a difference in efficacy of the treatments; small trials frequently conclude 'there was no significant difference', irrespective of whether there really is a treatment effect or not. In statistical terms, we would say that the 'sample size' is too small and that the 'power of the test' is very low.

The 'power' of a significance test is a measure of how likely a test is to produce a statistically significant result, given a true difference between treatments of a certain magnitude.

### Effect of sample size upon interpretation of significance

Suppose the results of a treatment difference in a clinical trial are declared 'not statistically significant'. Such a statement only indicates that there was insufficient weight of evidence to be able to declare that the observed data are unlikely to have arisen by chance. It does not mean that there is 'no clinically important difference between the treatments'. If the sample size was too small, as discussed in the previous paragraph, the study might be very unlikely to obtain a significant *P*-value even when a clinically relevant difference is present. Hence, it is of crucial importance to consider sample size and power when interpreting statements about 'non-significant' results. In particular, if the power of the test was very low, all one can conclude from a non-significant result is that the question of treatment differences remains unresolved; the study has provided little information of value. This has led some authors (e.g. Newell, 1978; Altman, 1980) to question whether studies with a too small sample size may be scientifically useless and hence an unethical use of subjects and other resources. Clearly, where possible, it is wise to aim at conducting a realistically sized trial. However, the role of small trials in scientific research remains important, albeit controversial, and we shall return to this issue below.

### *Example*

In 1985 the UK Medical Research Council (MRC) designed a randomised clinical trial (ST01) which compared two types of surgery for operable gastric cancer. The aim of this trial was to compare conventional surgery (R1), as widely practised in the West, with radical surgery including extended lymph node dissection (R2), which is commonly practised in Japan. The principal end point of interest was survival; reports from Japan showed that patients undergoing R2 surgery had appreciably longer survival durations than those experienced by patients in other countries, and attributed the difference to the surgery. Since R2 surgery is far more extensive and aggressive than R1, increased post-operative morbidity and possibly mortality would have to be offset by reasonably large survival advantages for R2 to be worthwhile.

Based upon past experience, it was estimated that the baseline survival rate of patients undergoing an R1 resection would be 20% at 5 years. The surgeons also thought that R2 surgery might offer appreciable benefits, and a 14% improvement, to 34%, was thought realistic and worthwhile. It was decided that a *P*-value of $P<0.05$ would be an acceptable 'significance level' for the test.

Thus, if a *P*-value of $P<0.05$ were obtained, we would be able to declare that such extreme data are unlikely to be due to chance, and that we believe there really is a treatment difference due to surgery; to be more precise, we would only expect such extreme results in one trial out of 20 (5%) purely by chance, and thus we would assume that R2 is more effective than R1.

Calculations show that 400 patients (185 per treatment arm, which was rounded up to 200) are required for a 90% power (Machin and Campbell, 1987). This indicates that, *if* R2 *does* improve survival by 14%, we would be 90% certain of obtaining a significance level of $P<0.05$; conversely, 10% of the time we would fail to obtain a 'significant' *P*-value (*P* not less than 5%), despite R2 being 14% better than R1. Sometimes this is described as a 10% false-negative rate. If

Correspondence: PM Fayers
Received 7 November 1994; revised 5 December 1994; accepted 6 February 1995

fewer patients were entered, we would be less likely to obtain a 5% $P$-value; thus 200 patients (100 per arm) would provide a power of 66%. In such circumstances, one-third of such trials could be expected to yield false-negative results. Such a low power is generally regarded as unacceptable. In most contexts, 80% is a realistic lower limit for power.

In 1993 the ST01 gastric cancer trial completed its intended patient accrual of 400 patients and the final results are awaited with interest.

### Size of cancer trials

It has long been recognised that many randomised clinical trials comparing therapies are unrealistically small. In 1978 Freiman *et al.* examined 110 'negative' trials, constituting approximately a third of all randomised controlled trials published in 20 different medical journals. Of these, 71 trials made explicit statements such as 'No significant reduction in mortality'. However, Freiman *et al.* showed that half of these trials had a power of less than 60% to detect a therapeutic benefit as large as 50%. They commented 'The conclusion is inescapable that many of the therapies discarded as ineffective after inconclusive 'negative' trials may still have a clinically meaningful effect'.

Yusuf *et al.* (1984), in a paper entitled 'Why do we need some large and simple randomised trials?', argued that for many new treatments, in many disease areas, it is only plausible to anticipate at best a relative mortality reduction of 15%; furthermore, especially in common diseases, even such modest mortality reductions are worth detecting since they imply the saving of many lives. Results from cancer trials over the decade since that paper strongly support the conclusion that in cancer, too, a major treatment breakthrough is frequently little more than a dream. The reality is that most therapeutic advances represent small steps, although collectively they may contribute to a larger overall improvement in survival. Few trials have demonstrated a mortality reduction as large as 15%.

Yusuf *et al.* also showed that the implication of this is that frequently trials ought to aim to enter many thousands of patients, and that even 2000 may be inadequate, although 'in real life, of course, the situation is even worse than this, as the average trial size is probably nearer to 200 than to 2000 patients!'. However, it is important to note that their paper relates to '*large, simple randomized* trials of the effects on *mortality* of various *widely practicable* treatments for *common conditions*'. This is often overlooked, although Freedman (1989) discussed areas in cancer research in which smaller trials (fewer than 1000 patients) would still be appropriate. These included common cancers with a high mortality rate, in which aggressive therapy would have to yield major survival improvements for the toxicity disadvantages to be outweighed, and areas where large trials are not feasible (rare cancers; treatments which can only be given in specialist centres). Over the years there has indeed been a gratifying increase in the average size of trials carried out by the principal clinical trials offices. For example, before 1990 the MRC Cancer Trials Office had conducted no trials with more than 1000 patients, whereas currently there are cancer therapy trials in colorectal cancer (AXIS trial, 4000 patients), ovarian (two ICON trials, each 2000 patients), prostate (PR06, 1800 patients), bladder (BA06, 1000 patients) and oesophageal cancer (OE02, 1000 patients), and even larger trials are being planned, e.g. of lung cancer (9000 patients). Protocols for these studies are available from MRC Cancer Trials Office.

The UK register of randomised cancer trials (Fayers and Armitage, 1993) currently lists 504 UK trials, and shows that 75% of trials which commenced before 1985 contained fewer than 430 patients. However, during 1985–89 this number had increased to 500 patients, and since 1990 it has increased again to 600 patients. However, there is no room for complacency. There are still many trials which appear to be initiated with scant regard for sample size. Since 1990 one in eight of the registered 98 trials has aimed for 50 or fewer patients per treatment arm.

### Reporting of trials

A well-designed trial will have formally estimated the required sample size and will have recorded the power calculations. Awareness of the importance of these has led to increasing numbers of medical journals demanding that full details be published with reports of trials. Thus the statistical guidelines for authors submitting papers to the *British Medical Journal* (Altman *et al.*, 1983) state 'Authors should include information on ... the number of subjects and why that number of subjects was used', and the accompanying check list for referees (Gardner *et al.*, 1986) asks 'was a pre-study calculation of required sample size reported?'. One important point arises from this last quote: the calculations should be made 'pre-study'. Some studies are initiated without adequate estimations of power and, especially when there is 'no significant difference', it is common for *post hoc* power calculations to be requested by referees and editors, or even by writers of subsequent correspondence. Unfortunately, as recently discussed by Goodman and Berlin (1994), 'Although several writers have pointed out the error implicit in the concept of post hoc power, such caveats have not had great impact'. The issues involved are subtle and rather complex, and to some extent remain controversial. The principal is that, whilst power calculations are vital to the design of a good study, they are of limited value and arguably useless to the subsequent interpretation of the single result that is observed. Therefore, while we require assurance that the study was well designed (pre-study power estimates), it is of little value to calculate power retrospectively. This arises from the logical inconsistency of applying pre-experiment probabilities which relate to a hypothetical group of results to a single result that is observed. The paper by Goodman and Berlin explains the problem in clear terms, and builds upon an analogy of 'trying to convince someone that buying a lottery ticket was foolish (the before experiment perspective) after they have hit the jackpot (the after-experiment perspective)'. Goodman and Berlin conclude 'avoid post hoc power estimates entirely', and recommend the use of confidence intervals and Bayesian estimates of treatment effects for study reporting.

Two points are incontestable. Firstly, sample size and power calculations should always be carried out before the investigation commences, with reports stating how and why they chose that number of subjects to study. Secondly, there is little value in a *post hoc* calculation which takes the form of, for example, 'we observed a difference of 14% which was not significant; however, the power of detecting a difference of 14% or greater for our sample size would have been <50%'; it can be shown that such statements are tautological, and the *post hoc* power corresponding to a non-significant difference is always <50%! (Goodman, 1992).

### Confidence intervals for interpreting and reporting results

The problems of interpreting power calculations in the context of observed differences can be largely avoided by greater use of confidence intervals. Investigators often have prior beliefs concerning the results that they expect to observe, and when a difference is not statistically significant such beliefs may manifest themselves by comments such as 'the result was not significant because there was too little power to detect small differences'. While it may be true that the study had little power to detect a real difference of the magnitude of the observed one, the apparent implication that the observed difference is a reliable indicator of the magnitude of the real one is unfounded. The observed difference is more suitably summarised by presenting confidence intervals, indicating the range of values within which the true treatment difference is likely to lie. This shows, far more informatively than comments about power, the impact of sample size upon the precision of an estimate of treatment effect. It is reassuring to note the increasing use of confidence intervals in medical journals, supplementing the use of $P$-values and reporting of power calculation.

### Estimation of sample size and power

Having discussed the importance of estimating sample size and power, we are led to the question of how best to do it, and the practical difficulties that arise. In this paper we choose to focus most of the discussion upon sample size considerations for a cancer therapy trial which is comparing two treatments with respect to differences in the survival of the patients. We do this because (a) such trials are especially common in the field of clinical cancer research and (b) most of the difficulties which are particularly apparent in this setting are equally valid in the context of other studies. Thus we assume a trial in which patients are randomly allocated either to a standard or control treatment, or to a new treatment. We also limit discussion to trials which are seeking to establish a treatment benefit; there are additional considerations to take into account when designing a trial which aims to establish treatment equivalence (Stenning and Altman, 1994). In estimating the number of patients required for a study (sample size), it is usual to identify a single major outcome which is regarded as the primary end point for comparing a treatment difference. In many clinical trials this will be a measure such as survival, response rate, time to relapse, degree of palliation or a quality of life index. Sometimes there is more than one outcome and all are regarded as of equal importance; this is dicussed below.

It is customary to start by specifying the size of difference that it is desired to detect, and then to estimate the number of patients that is required to enable the trial to detect this difference if it really exists. Thus, for example, it might be anticipated that a new treatment could improve 5 year survival from 20% of patients (old treatment) to 28% (new), and that, since this is a plausible and medically important improvement, it is desired to be reasonably certain of detecting such a large difference if it really exists. 'Detecting a difference' is usually taken to mean 'obtain a statistically significant difference with $P<0.05$'; and similarly the phrase 'to be reasonably certain' is usually interpreted to mean something like 'have a chance of at least 90% of obtaining such a $P$-value' if there really is an improvement from 20% to 28%. The last statement corresponds, in statistical terms, to saying that the power of the trial should be 0.9. It is common to design trials requiring a power of 80% or 90%. For a survival study, providing the survival curves are likely to be approximately exponential, these four details (proportion of survivors in the control arm, difference in survival proportions, significance level, power) suffice to enable the required sample size to be estimated.

### Number of patients or number of deaths?

When designing a clinical trial, it is customary to express the size requirements of the trial in terms of the number of patients that it is desired to recruit. However, the required size is really determined by the number of 'events' (for example, deaths) that arise. To see that this must be so, consider the following two extreme situations. Firstly, suppose all patients have been recruited to the trial, but that no deaths have occurred; clearly we cannot estimate the relative survival rates, and have no information upon which to base the comparison of treatment groups. Secondly, suppose all patients have been recruited, and that all have died; in this case, we have full information about the survival curves for the patients entered into the trial, and their possible treatment-related differences. Hence the information about the survival curves is contained in the deaths ('events'), and the objective in designing a trial with a large number of patients is simply to increase the number of deaths that we expect to observe.

The mathematical equations which enable us to estimate the number of patients required in a survival study are, therefore, based upon first estimating the number of events required; the number thus obtained is then used to calculate the number of patients expected to produce these events, by assuming that patients enter the study in a systematic manner and die at a steady rate. The distinction between patients and events is important, and one that is often overlooked when simply reading off 'number of patients required' from sample size tables. Many protocols, too, merely state the number of patients required in order to be able to detect the target difference with reasonable certainty, and omit to indicate that the number of patients has been derived indirectly and is based upon having calculated the number of deaths that were estimated to be necessary.

*Example*

Calculations for the MRC ST01 trial, based upon the details given earlier, indicate that 135 'events' per treatment group are required in order to obtain the desired power. The statement '400 patients (200 per treatment arm) are required' was an estimate of the number of patients that is required in order to accrue 270 deaths by the time the data will be analysed.

### Timing of analysis

Important consequences arise from the distinction between number of patients and number of events. Even though the specified number of patients may have been recruited to the trial, the intended power will only be attained by waiting until the required number of deaths have accrued. Thus, the analyses for a clinical trial designed to have a particular power level should take place at a particular time; too soon, and the lack of events will cause a loss of power; too late, and unnecessary extra power will be yielded. Also, if fewer deaths were observed than anticipated, either the sample size should be increased by entering additional patients or analysis of the results should be delayed until the necessary deaths are observed.

Most tables for the number of patients in a log-rank test (e.g. Freedman, 1982; Machin and Campbell, 1987) assume that the patient accrual is at a constant rate, so that the median length of follow up is equal to one-half the accrual period plus the length of the post-accrual period (Freedman, 1982; Haybittle *et al.*, 1990).

In practice, there will be an accrual period and then a follow-up period; usually one estimates the baseline survival rate and the anticipated survival rate for the treatment groups as the expected survival probabilities for a given median length of follow-up at the time of the intended analyses. In the case of a trial involving treatment for poor-prognosis disease, the median duration of follow-up may be short since the events happen rapidly. However, the MRC has this year launched a trial (PR06) in early prostate cancer in which the prognosis is favourable; therefore analyses will have to be deferred until patients have a median follow-up of 10 years, at which time 75% or more of patients are still expected to be alive and metastases free.

*Example*

Interim analysis of the ST01 trial suggested that the baseline (R1) survival may be 27% at 5 years, instead of 20% as expected initially; thus fewer events would be accrued by the planned date of analysis, which was at 5 years' median duration of follow-up. There are several reasons why this might be so: not all patients are entered into clinical trials, and those recruited to ST01 may have been healthier than anticipated; medical care may have changed over the years; cases may be diagnosed earlier. However, as is often the case with survival studies, at the time of the interim analysis few patients had reached 5 years and so the estimate of 27% was based upon very small numbers. Hence, the estimate had a wide confidence interval, and a true rate of 20% was still plausible. In the event, the steering committee for this trial decided not to continue patient accrual beyond the planned 400. Instead, if necessary, analysis of ST01 will be delayed until sufficient deaths have been accrued.

Suppose, however, that 27% is the true baseline survival rate, and that the data would be analysed at the time planned. Calculations show that the power to detect 41% survival rate (absolute percentage improvement of 14%) in the R2 arm would have fallen to 87%. Such a small reduction in power is probably unimportant, but if it was really felt necessary to maintain 90% power the recruitment could have been extended to 450 patients.

### Precision

We have already indicated that, to estimate sample size for a survival study, one must specify the baseline survival rate, the treatment difference that one seeks to detect, the significance level for the test and the desired power of the test. But all these variables either can be difficult to determine with precision or are totally subjective. There is nothing special about a 5% *P*-value, as opposed to a 5.1% or a 4.9% *P*-value; and the distinction between 90% power or 89% power is likely to be of little practical relevance. Similarly one might, for example, specify that a trial should be able to detect a 20% difference in median survival; presumably a 19% difference is of almost equal interest, and the specification of 20% is therefore a purely arbitrary value. Thus, it is of interest to conduct a 'sensitivity analysis', which can show the effect of varying the initial requirements. Some of the better computer programs for sample size estimation provide graphical support for displaying the results of sensitivity analysis.

Here, however, we will focus upon one particular aspect, namely sensitivity analysis of the difference that it is desired to detect. Suppose 10% of patients are expected to survive to 5 years, and we wish to detect an improvement to 20%. Figure 1 shows that we would require approximately 200 patients per group. But suppose we decide this is an unreasonably optimistic difference to seek – few chemotherapy trials seem to produce so great an improvement; perhaps 17.5% is a more realistic target. Now we need over 300 patients per group (since $p_1 - p_2 = 0.075$). Or maybe 15% ($p_1 - p_2 = 0.05$) should be the target: 600 patients per treatment group! Thus, it is apparent that even small changes in the input variables can result in dramatic variations in the sample size estimates. It is rarely of any relevance to quote a precise estimate of the number of patients required, and it is customary to liberally round any estimates upward, as was done in the ST01 example when 185 patients per group was rounded up to 200 patients.

**Figure 1** Sample sizes required to detect survival differences $p_1 - p_2$, for 90% power and 5% *P*-value.

### Size of differences

The estimated sample size that is required might be written in a statement such as 'to be 90% certain of detecting a treatment difference of *dd* or greater, with a 5% significance level, we require … patients'. Thus, in order to estimate sample size and power, one must first identify the magnitude of the difference that it is wished to detect.

Sometimes there is prior knowledge which enables an investigator to predict what treatment benefit is likely to be observed, and the role of the trial is to confirm the expectations. At other times it may be possible to say that, for example, only a doubling of median survival would be worthwhile because the new treatment is so toxic and expensive. In such cases the investigator may have definite opinions about the treatment difference that is pertinent to detect. However, very frequently, there is no obvious specific treatment benefit that is sought. It may be that even a small treatment benefit is regarded as important; for example, in survival studies any improvement in survival rates may save many lives if the disease is a common one. Thus, there will have to be discussion about the magnitude of detectable differences and the sample size. One suspects that in practice a form of iteration is often used. A clinician might specify a clinically useful difference that it is hoped could be detected, and would then estimate the required sample size. Perhaps we wish to be reasonably certain of obtaining a 'significant *P*-value' if the survival advantage to a new treatment is 5% at 2 years. The calculations might then indicate that an extremely large number of patients is required. The investigator may next say 'suppose we aim to be reasonably certain of detecting a rather larger difference, say 10%'; the calculations are repeated, and perhaps the sample size is still too large for the trial to be feasible. Perhaps the investigator is willing to accept a power of 80% as being 'reasonably certain of obtaining a 5% *P*-value'; the calculations are repeated again. Also, the investigator might say: 'we can only recruit 500 patients within a reasonable number of years; what differences can we detect with that sample size?'. Eventually, by iteration and compromise, either there is agreement concerning the number of patients and the differences that can be detected or the investigation must be deemed not feasible.

Some clinical trial protocols openly indicate their uncertainty; thus the MRC head and neck cancer protocol (CHART, protocol available from MRC Cancer Trials Office) states: 'we require 460 patients to detect an improvement from 45% to 60%, 263 patients to detect an improvement from 45% to 65% … Given these considerations (and assuming a loss to follow-up of 10%) we shall aim to recruit 500 patients into this study'.

One additional problem is that investigators are often optimistic about the effect of new treatments; it can take considerable effort to initiate a trial, and so in many cases the trial would only be launched if the investigator is enthusiastic about the new treatment and is sufficiently convinced about its potential efficacy. The experience of many trials offices would seem to be that as trials progress there is often a growing realism that, even at best, the initial expectations were optimistic. To some extent clinicians' optimism is likely to be tempered by the cynicism of statisticians in trials offices, for there is ample historical evidence to suggest that trials which set out to detect large treatment differences nearly always result in 'no significant difference was detected'; in such cases there may have been a true and worthwhile treatment benefit, but the level of detectable differences was set unrealistically high and thus many trials have been underpowered.

Another issue, discussed later, is the crucial distinction between the plausible differences that might be present and the minimally worthwhile difference.

### *Example*

The MRC trial of gastric surgery, ST01, was designed by Laurence Freedman in 1985, and is exceptional in the care that was taken in its sample size estimation. Eight surgeons and clinicians, who were experienced in the treatment of gastric carcinoma, were individually interviewed, and another 18 were sent postal questionnaires. They were asked about what they expected to be the survival rate in the control group, what difference in survival might be anticipated for the aggressive alternative therapy and what difference would be medically important and could influence their future treatment of patients. Few other trials have been designed with such care. On the basis of the opinions expressed, it was decided to seek to detect a change in 5 year survival from 20% (R1 surgery) to 34% (R2 surgery). However, it has become apparent that over the last 7 years surgeons have modified their views about R2 surgery, and now believe that 14% difference is too optimistic. Therefore, before the trial was closed and before any survival information was revealed to the steering committee, there was discussion of what difference might be plausible and yet still worthwhile. The prevailing consensus opinion is now that a 10% or even 8% difference would be more realistic, and that this would still represent a sufficiently large difference to influence future surgical practice. If the calculations are repeated on the basis of 27% survival in R1 and 37% in R2 (an improvement of 10%), over 800 patients would be required. An improvement of 8%, to 35% in R2, would require 1260 patients. This is more than three times as many patients as in the ST01 trial, and at present recruitment rates would require another 14 years of patient accrual! In the event, it was decided not to extend patient recruitment; a parallel trial was conducted in The Netherlands, and a meta-analysis of the two trials is planned.

### Is sample size estimation worthwhile?

We have discussed a number of problems in deciding what values to use for baseline survival and worthwhile detectable differences, and have shown how sample size and power calculations are greatly influenced by the precise values used. Also, the ST01 trial is an example of how even the most carefully planned trials may be based upon estimates which with hindsight are very suspect – even though they were based upon the best available information at the time the trial was designed. One might therefore be tempted to question the value of sample size estimation – is it ever possible to obtain meaningful estimates of sample size? Fortunately, however, this trial is in many ways atypical and has been deliberately chosen so as to highlight the potential problems. Frequently there will be prior information and past experience concerning baseline survival rates and the likely difference that might apply to the new treatment. As discussed below, when such information is not available, pilot studies may offer one way forward. Also, by calculating sample sizes for a variety of plausible baseline estimates and differences, it is possible to obtain an idea as to whether the proposed study is likely to be unrealistic.

In all cases, however, it is important to make the best estimate that one can when planning a trial; the points that we wish to emphasise in summary are that (a) it is futile to regard estimates of sample size as precise when there is so much uncertainty about the survival rates – they should be treated with caution and usually rounded upwards; (b) sensitivity analysis can be revealing at the design stage, and power implications should also be reviewed at later stages in the trial and when it is analysed; and (c) one should be circumspect about the whole procedure of estimating sample size. Despite these difficulties, however, it is essential to perform sample size calculations before the start of a trial.

### Assumptions underlying sample size estimates

Estimates of sample size and power are based upon similar assumptions to significance tests; in fact, in order to be able to make these estimates it is necessary to specify in advance the methods of analysis that will be used. Thus for survival analysis it is necessary to specify, before performing the sample size calculations, what significance test will be used. Two commonly used methods of analysis are the log-rank test and tests based upon Cox or similar models. The Cox model explicitly assumes what is known as exponential survival curves with proportional hazards; however, although the log-rank test does not make the same assumptions, the estimate of power associated with the log-rank test does depend crucially upon the same assumptions. Thus, it is important to consider whether these assumptions are valid for any particular study and what the effect is of violation of assumptions. Particular examples of violation might be: (a) survival curves in which the hazard rate changes, for example where there is an initial high post-operative risk following surgery, or a reduced death rate for the duration of a chemotherapy treatment, followed by a different death rate at later stages; (b) survival curves might cross over, for example when initial aggressive therapy causes early deaths but improves survival rates in later years; (c) if one treatment 'cures' a proportion of patients, while other patients continue to die according to the initial death rate. In such cases the log-rank test may not be optimal and the Cox model is likely to be inappropriate; sample size estimates will need to take into account the departures, which will frequently necessitate a larger sample size.

It seems likely that the fundamental assumptions are frequently violated. Minor departures may have little effect upon power. If a major deviation is anticipated, there should be an adjustment to the sample size estimates. Various models have been proposed, each dealing with particular types of departure from assumptions. Often, however, there is insufficient prior information about the precise shape of the survival curves, and adjustments to sample size rely greatly upon statistical experience.

### *Example*

In the MRC ST01 trial, the consensus surgical view is that the survival rates following R1 surgery are likely to be approximately exponential, but following R2 surgery there will initially be a higher risk of post-operative mortality, followed by an exponential survival curve until about 3–4 years, and finally those patients who reach 4 years are likely to have been 'cured', which will cause the R2 survival curve to flatten out and cross the R1 curve. However, the R2 survival curve remains highly speculative and it is difficult to use these prior expectations for power calculations.

### Nomogram

One very simple means of estimating sample size while also obtaining a feel for the sensitivity of the results to variations in the specified factors is to use a nomogram. Figure 2, described in the appendix, presents a nomogram for the log-rank test. It is very easy to perturb a straight edge so as to see the effect of varying the hazard ratio or the baseline hazard. It is also difficult to read an exact value for sample size from a nomogram; we would maintain that this inability is an advantage over using tables, in that it forces one to be more aware of the inherent imprecision of the numerical estimates. A nomogram provides as much precision as is appropriate for designing a trial. Finally, a nomogram like this summarises onto one sheet the equivalent of several pages of tables.

### More than one primary outcome

It is implicit in the above discussion that there is a single identifiable end point or outcome upon which treatment comparisons are based. Sometimes there is more than one end point of interest, such as survival, response rates and quality of life scores. If one of these end points is regarded as

**Figure 2** Nomogram for the power of the log-rank test (two-tailed test).

more important than the others, it can be named as the primary end point and sample size estimates calculated accordingly. A problem arises when there are several outcome measures which are all regarded as equally important. A commonly adopted approach is to repeat the sample size estimates for each outcome measure in turn, and then select the largest number as the sample size required to answer all the questions of interest.

Here, as with the discussion of violation of significance test assumptions, it is essential to note the relationship between significance tests and power: it is well recognised that *P*-values become distorted if many end points are each tested for significance, and that adjustments should be made. Often a smaller *P*-value will be considered necessary. In such cases, the sample size calculations will be similarly affected.

A similar situation may be observed in a clinical trial which compares three or more treatments; two common methods of analysis are either to consider all pairwise comparisons (for example, four treatment arms would result in six pairwise comparisons), or to use a global statistical technique such as analysis of variance. In either case, the sample size calculations should reflect the intended method of analysis.

### Are small trials of any value?

In the context of this paper, a 'small trial' refers to one which is too small to have a reasonable chance of detecting any plausible difference, that is a trial with low power and which is likely to yield a non-significant *P*-value even when the hypothesised treatment difference really exists. On the face of it, such small trials are worthless. There are, however, two opposing schools of thought. At one extreme, it can be claimed that, whenever a clinician has any doubts about the merits of two alternative treatments, the allocation should be randomised. It is always better to conduct a randomised trial and try to gain more scientific knowledge about the treatments than to let patients be treated in a haphazard manner according to the clinicians' whims. It is often suggested that small trials are better than no trial at all, because their results may well be of value when combined with data from other sources, as in an overview or meta-analysis. At the other extreme, however, the counter-argument maintains that most improvements in cancer therapy are in themselves small steps, and small trials will never be able to detect such differences. Small trials are therefore doomed to obtain misleading results, claiming 'no difference' even when a potentially very useful treatment advantage is in fact present. Furthermore, if a small trial *does* obtain a significant *P*-value, the estimate of the treatment difference will almost certainly be an overestimate, often a gross overestimate, but with a wide confidence interval.

We hold a more mixed view. It is always preferable to aim for a trial that has a reasonable chance of obtaining a meaningful result, that is to say a trial which has adequate power to detect the difference of interest. However, if this is not practicable, we would accept that any trial is better than no trial, provided two conditions are met:

(1) All publications about the trial must make it clear that the power was low, and that the results can at best be regarded as hypothesis forming. Deeper interpretations cannot be place upon either significance or non-significance, even though the temptation is to be dismissive of non-significance ('What can one expect – the sample size was too small') and to attach too much importance to significance (in a small trial, lacking power, a value of $P<5\%$ will frequently indicate no more than that the trial is among the 5% which one expects to return false-positive *P*-values).
(2) The trial, like all trials, should be registered before it is commenced (Fayers *et al.*, 1993; Fayers, 1994) so that, even if it fails to be subsequently published, the results will be known and available for use in overview or meta-analyses. The existence of publication bias has been well established (Simes, 1986; Dickersin *et al*., 1987; Newcombe, 1987; Begg and Berlin, 1988; Dickersin and Min, 1993); large trials are likely to be published, whereas small trials are more likely to be published only if they have 'significant' results. Hence preregistration of trials is essential.

**Fewer control patients**

Sometimes, either becasue the total number of patients is limited or because it is thought that the new treatment is definitely superior, there have been attempts to reduce the number of patients in the control arm of a trial. The basic arguments in favour of randomised trials and against studies based upon historical controls are old and well known (Chalmers *et al*., 1977; Pocock, 1983; Sacks *et al*., 1983; Gehan, 1984; Micciolo *et al*., 1985; Diehl and Perry, 1986). In essence, historical controls carry risks of serious bias owing to possible patient selection bias, differences in response criteria and changes over time. The references cited also contain examples in which studies using historical controls have subsequently been shown to have resulted in biased conclusions in favour of the new treatments. Of more interest, however, is the work by Pocock (1976, 1983), which considers using a mixture of historical controls and randomised controls, with more weight being given to the latter group. The idea here is to use an unequal randomisation, in which the majority of patients are randomised to the new treatment, and to supplement the fewer randomised controls by combining them with the historical controls. Unfortunately, the same inherent problems about historical controls still apply: not only will the sample size be too small to permit a sensitive comparison between historical controls and randomised controls, but there remains the possibility that the historical controls are not representative of current patients and therefore seriously biased. Perhaps for these reasons, such schemes do not appear to have been widely adopted in clinical trials.

It is customary to design clinical trials with equal numbers of patients in the two treatment arms; this is nearly optimal in terms of obtaining maximum power for a given total sample size. (The perceptive reader might note that since survival comparisons obtain their information from deaths, not patients, it would in fact be preferable to weight the allocation ratio towards equal numbers of expected deaths in the two treatment arms! However, under the null hypothesis, we assume equal death rates for the two treatment groups.) Although we would not recommend use of historical controls, there may be many situations in which an unequal randomisation ratio could be of value. It can be shown statistically that if, for example, the randomisations are weighted so that for every three patients allocated to the new treatment there are two allocated to the control group, there is very little impact upon the power to detect treatment differences; by only slightly increasing the total sample size it is possible to maintain the same power as for equal allocation ratios. Hence, if it is thought that there are reasons to avoid using the control therapy (such as serious side-effects), it is possible, with little increase in sample size, to allocate the greater proportion of patients to the new therapy. One example of such a trial is the MRC BR06 protocol (available from MRC Cancer Trials Office), which used a 2:1 allocation ratio. This was done because primary CNS lymphoma is a relatively rare cancer, and it was decided that there was already sufficient knowledge about the standard treatment, radiotherapy, but that greater information was required about adjuvant chemotherapy.

**Pilot studies**

In order to calculate the power or sample size of a trial, one must first have available background information; for example, for a survival analysis one must know the expected survival of the control or baseline arm. Also, one must have some idea as to what is a realistic difference to seek. Sometimes such information is available as prior knowledge; at other times, a pilot study may be conducted.

Traditionally, a pilot study is a distinct preliminary investigation, conducted before embarking on the main trial. Recently, however, Wittes and Brittain (1990) have explored the use of an internal pilot study. The idea here is to plan the clinical trial on the basis of best available information, but to regard the first patients entered as the internal pilot. When data from these patients have been collected, the sample size can be re-estimated with the revised knowledge. Two vital features accompany this approach: firstly, the final sample size should only ever be adjusted upwards, never down; and, secondly, the authors clearly explain why one should only use the internal pilot in order to improve the estimation of factors which are independent of the treatment variable. This second point is crucial. It means that for a $t$-test one might estimate the variance, or for a survival comparison one might estimate the survival of the control arm; in neither case should the sample size be adjusted because of any apparent differences between treatments which might be observed during the pilot phase. The reasons for the first point, only ever adjusting upwards, are rather more subtle, but nonetheless important. Both these points should be carefully observed, however, to avoid distortion of the subsequent significance test.

The advantage of an internal pilot is that it can be relatively large – for example, half the anticipated patients – with no increase in time or money. It provides an insurance against misjudgement regarding the baseline assumptions. It is, however, important that the intention to conduct an internal pilot study is recorded at the outset and that full details are given in the study protocol; otherwise there may be suspicion that the investigators performed multiple looks before deciding to make *ad hoc* changes to the protocol.

The theoretical implications of this approach are still being explored, but it would appear to place on a more scientific footing a procedure which one suspects has sometimes been instinctively yet covertly applied (and misapplied) by triallists in the past.

**Clinician's prior beliefs**

The usual approach to sample size estimation requires specification of the size of difference that it is intended to detect. However, this forces the clinician into a rather artificial situation in which there is a simple cut-off where one difference is regarded as realistic and of medical interest, whilst a slightly smaller value is classified as of being of no interest. Furthermore, it is likely that other clinicians would have very different views as to what is the critical value of interest. Spiegelhalter and Freedman (1986) have pioneered a method of assessing clinicians' prior beliefs and expectations. A fundamental aspect of this approach is that the null hypothesis is no longer simply 'no difference' between the treatments, but is clinical equivalence as determined by interviewing the clinical participants. Specifically, participants and other experts are asked 'what differences in survival rate would influence you to use treatment 1 or treatment 2?, and could choose a range of values within which they would remain uncertain which treatment to use; if, however, the difference was more extreme in one direction they would prefer to use treatment 1, whilst if it was more extreme than the equivalence range in the other direction they would choose treatment 2. This probably reflects clinical thinking more closely than if one demanded a single value for the treatment difference, above which treatment 1 is preferred and below which treatment 2 is favoured. This approach was used in the MRC gastric trial, and most clinicians indicated that a 5–10% 5 year survival advantage to R2 would leave them uncertain whether to use R1 or R2 surgery (because R2 surgery is accompanied by worse morbidity); some chose 10–15% as equivalent, and a few chose 10–20%.

The clinicians were also asked to indicate what difference in survival they expected would emerge if many patients were given the two treatments; they could indicate a range of values, and weight their beliefs. For example, in the MRC gastric trial, a clinician might have indicated that the 5 year survival advantage to R2 surgery was probably more than 20%, and that it could be, but was less likely to be, above 25%, and that it could even be 30% or more, although this would be even less likely.

Spieglehalter and Freedman show that the information collected in this manner can be used to examine how different sample sizes affect the chance of reaching a firm conclusion from the trial, which they call the 'predictive power' or 'strength' of the trial. This emphasises one often overlooked aspect of clinical trials, namely that the role of a clinical trial should not be merely to establish treatment differences, but should be to influence medical practice.

This method has proved extremely valuable for elucidating clinicians' opinions and obtaining a general feel as to what is the difference of interest. The authors noted that 'the clinicians frequently voice the opinion that, although a difficult exercise, it made them think deeper about the forthcoming trial than they had done previously'. Unfortunately, it is more difficult to incorporate the method into a formal procedure for producing a single estimate of sample size, and its value is rather more to enable graphical methods to display the range of opinions and their effects upon sample size. More recent work by Spiegelhalter *et al.* (1993) has extended these interesting ideas.

### Sequential trials

This article discusses conventional or 'fixed sample size' trial designs. There are a number of other approaches which are based upon sequential or repeated analyses of the accruing data in the trial. Such designs do not specify a single sample size, but typically use a predefined stopping rule; patients continue to be entered until there is sufficient evidence either that there is a treatment superiority or that there is unlikely to be any difference between the treatments (e.g. Whitehead, 1992). Sequential designs may be appropriate when it is important to terminate a trial at the earliest possible opportunity, either for ethical reasons (for example, to avoid unnecessary deaths) or for financial reasons (cost of one treatment arm). However, they depend upon having a reasonable proportion of outcome results at the time of conducting each interim analysis. For example, it would clearly be impractical to consider a sequential scheme if the outcome of interest is 5-year survival whilst the expected accrual period for patients is only 3 years. Sequential designs also depend upon having a single value of clinical importance, whereas in practice there is frequently more than one.

Sequential designs remain controversial and are less widely used than fixed sample size trials, possibly partly because of their additional mathematical complexity and the need to use computer software for the calculations. Some of the issues involved are discussed by Fayers *et al.* (1994).

### Prognostic factors

Sample size calculations for prognostic factors studies must depend very much upon the method of analysis that will be used for the modelling. However, many authors have noted that far too often prognostic factor studies are absurdly small, especially those in which survival is the outcome to be predicted (e.g. Simon and Altman, 1994). Also, as always with Cox models, sample size relates to the number of events observed and not the number of patients; if the event rate is low, the total number of patients must be increased accordingly.

Simon and Altman (1994), in an earlier editorial, have discussed statistical aspects of prognostic factor studies. As they note, when the sample size is too small, there are problems of multiplicity of testing which frequently result in potential predictor variables being declared significant by chance alone. The relative importance of the 'significant' factors will also be unreliable. Claims for the predictive accuracy of the prognostic equation are liable to be grossly overstated. That a small sample size is a real practical problem can be readily observed: if one compares the many published papers to be found describing prognostic factors within a single disease area, it is common to find major divergence of opinion as to the most useful factors – and even as to which is the most important single factor.

Very little work has been done upon formal methods of estimating sample sizes when evaluating prognostic factors, although various authors have suggested rules based upon intuition and experience. For example, Harrell *et al.* (1985) suggest that, with half the data set being used for 'training' and the other half reserved for subsequent validation of the prediction equation, then 'as a rough rule of thumb one should not attempt a stepwise (Cox) regression analysis when there are fewer than ten times as many events in the training sample as there are candidate predictor variables. The problems become more severe when one considers interaction terms in the mode.'

Alternatively, Fielding *et al.* (1992) suggest that the procedure for introducing a new candidate factor into existing prognostic models should be 'first ... the prognostic relationship will be evaluated in a study of several tens of patients (e.g., 50–100). If the results appear promising ... the results will be studied on several hundreds of patients.' They also note that, after a statistical model has been developed, its validity should be verified from a separate data set; this requires yet more patients.

A paper by Schumacher *et al.* (1994) investigates the use of Cox models for evaluation of prognostic factors. They note that prognostic factors should exhibit large relative risks if they are to be useful, which might at first sight suggest that smaller numbers of patients are required. Practical experience combined with the results from their simulation studies lead them to suggest that 'studies with less than 25 events per factor cannot be considered as an informative and reliable basis for the evaluation of prognostic factors.' Furthermore, the relative risks and prevalence of each factor must be considered. They conclude that small studies can at best only serve to create exploratory hypotheses, and might lead to misleading confusion; the large studies that are necessary will often require collaboration between groups, or the use of meta-analyses.

Schumacher *et al.* also describe a formula for sample size when considering a single binary prognostic factor. However, prognostic factor studies invariably involve a number of factors, often including some with more than two levels. This necessitates using a general multivariate form of the simpler equation, and to solve this one would have to know the multivariate distribution of the prognostic factors in advance, which is not realistic (M Schumacher, personal communication). Thus, to a large extent one has to rely upon experience, supported by simulation and some theory for some typical situations.

### Conclusions

Sometimes the estimation of sample size can be based upon precise requirements accompanied by detailed information about baseline rates and variability. Unfortunately, in our experience, such situations are rare. All too often in clinical trials and many other medical investigations there is a lack of prior knowledge about what to expect from the study, making sample size calculations fraught with difficulty. However, despite the attendant problems, the estimation of sample size and the consideration of power implications is of fundamental importance to the design of a sensible and realistic study and should always be undertaken with the greatest of care. Full details of the methods used to estimate sample size requirements should be recorded. We note with approval that such information is increasingly demanded by funding bodies, independent protocol review committees, ethical review panels, and, at the conclusion of the study, journals to which reports are submitted.

### Appendix A: Use of the nomogram

The left scale gives the power of the log-rank test corresponding to $P<0.01$ and $P<0.05$. The middle scale gives the total number of deaths or events that it is required to observe. The right scale shows the change in survival, presented as a percentage change in the median or as the hazard ratio. If a straight edge is placed over selected values on any two of the scales, the corresponding value may be read off the third one.

For example, suppose we wish to detect a change from a median survival of 10 months to a median survival of 15 months, and choose to use a power of 90% and a 5% $P$-value. The change in median survival corresponds to a 50% increase. Reading across the nomogram suggests that the appropriate sample size is 260 events, or 130 per treatment group. If a power of 80% is acceptable, a total of 180 events would be required, whilst for 90% power and a 60% increase in median survival just under 200 events are required.

Alternatively, if survival proportions $\pi_1$ and $\pi_2$ are used instead of medians, the hazard ratio is given by $h = \log(\pi_1)\ \log(\pi_2)$; and this is also shown on the right-hand scale.

If $\pi$ is the average survival proportion $\pi = (\pi_1 - \pi_2)\ 2$, then for $e$ events the number of patients can be estimated by $e\ (1 - \pi)$.

### References

ALTMAN DG. (1980). Statistics and ethics in medical research. III. How large a sample? *Br. Med. J.*, **281**, 1336–1338.

ALTMAN DG, GORE SM, GARDNER MJ AND POCOCK SJ. (1983). Statistical guidelines for contributors to medical journals. *Br. Med. J.*, **292**, 1489–1493.

BEGG CB AND BERLIN JA. (1988). Publication bias: a problem in interpreting medical data. *J. R. Stat. Soc.*, **151**, 419–463.

CHALMERS TC, MATTA RJ, SMITH H AND KUNZLER A-M. (1977). Evidence favouring the use of anticoagulants in the hospital phase of acute myocardial infarction. *N. Engl. J. Med.*, **297**, 1091–1096.

DICKERSIN K AND MIN YI. (1993). NIH clinical trials and publication bias. *Online J. Curr. Clin. Trials*, **April 28**, Document No. 50.

DICKERSIN K, CHAN S, CHALMERS TC, SACKS HS AND SMITH H. (1987). Publication bias and clinical trials. *Controlled Clin. Trials*, **8**, 343–353.

DIEHL LF AND PERRY DJ. (1986). A comparison of randomized concurrent controlled groups with matched historical controls groups: are historical controls valid? *J. Clin. Oncol.*, **4**, 1114–1120.

FAYERS PM. (1995). The UKCCCR Register of UK Trials. *Clin. Oncol.* (in press).

FAYERS PM AND ARMITAGE T. (1993). Towards an international register of cancer trials: the UKCCCR Register of UK Trials. *Eur. J. Cancer*, **29A**, 907–912.

FAYERS PM, COOK PA, MACHIN D, DONALDSON N, WHITEHEAD J, RITCHIE A, OLIVER RTD AND YUEN P. (1994). On the development of the Medical Research Council trial of α-interferon in metastatic renal carcinoma. *Stat. Med.*, **13**, 2249–2260.

FIELDING LP, FENOGLIO-PREISER CM AND FREEDMAN LS. (1992). The future of prognostic factors in the outcome prediction for patients with cancer. *Cancer*, **70**, 2376–2377.

FREEDMAN LS. (1982). Tables of the numbers of patients required in clinical trials using the logrank test. *Stat. Med.*, **1**, 121–129.

FREEDMAN LS. (1989). The size of clinical trials in cancer research – what are the current needs? *Br. J. Cancer*, **59**, 396–400.

FREIMAN JA, CHALMERS TC, SMITH H AND KUEBLER RR. (1978). The importance of Beta, the type II error and sample size in the design and interpretation of the randomised control trial. *N. Engl. J. Med.*, **299**, 690–694.

GARDNER MJ, MACHIN D AND CAMPBELL MJ. (1986). Use of check lists in assessing the content of medical studies. *Br. Med. J.*, **292**, 810–812.

GEHAN EA. (1984). The evaluation of therapies: historical control studies. *Stat. Med.*, **3**, 315–324.

GOODMAN SN. (1992). A comment on p-values, replication and evidence. *Stat. Med.*, **11**, 875–879.

GOODMAN SN AND BERLIN JA. (1994). The use of predicted confidence intervals when planning experiments and the misuse of power when interpreting results. *Ann. Intern. Med.*, **121**, 200–206.

HARRELL FE, LEE KL, MATCHAR DB AND REICHERT TA. (1985). Regression models for prognostic prediction: advantages, problems, and suggested solutions. *Cancer Treat. Rep.*, **69**, 1071–1077.

HAYBITTLE JL, ALCOCK CJ, FOWLER JF, HOPEWELL JW, REZVANI M AND WIERNIK G. (1990). Recruitment, follow-up and analysis times in clinical trials of cancer treatment: a case study. *Br. J. Cancer*, **62**, 687–691.

MACHIN D AND CAMPBELL MJ. (1987). *Statistical Tables for the Design of Clinical Trials*. Blackwell Scientific Publications: Oxford.

MICCIOLO R, VALAGUSSA P AND MARUBINI E. (1985). The use of historical controls in breast cancer. *Controlled Clin. Trials*, **6**, 259–270.

NEWCOMBE RG. (1987). Towards a reduction in publication bias. *Br. Med. J.*, **295**, 656–659.

NEWELL DJ. (1978). Type II errors and ethics. *Br. Med. J.*, **2**, 1789.

POCOCK SJ. (1976). The combination of randomized and historical controls in clinical trials. *J. Chron. Dis.*, **29**, 175–188.

POCOCK SJ. (1983). *Clinical Trials – A Practical Approach*. John Wiley: Chichester.

SACKS HS, CHALMERS TC AND SMITH H. (1983). Sensitivity and specificity of clinical trials. *Arch. Intern. Med.*, **143**, 753–755.

SCHUMACHER M, SCHMOOR C AND SAUERBREI W. (1994). Evaluating the impact of prognostic factors on survival times: sample size considerations, preprint. Albert-Ludwigs-University: Freiburg.

SIMES RJ. (1986). Publication bias: the case for an international registry of clinical trials. *J. Clin. Oncol.*, **4**, 1529–1541.

SIMON R AND ALTMAN DG. (1994). Statistical aspects of prognostic factor studies in oncology. *Br. J. Cancer*, **69**, 979–985.

SPIEGELHALTER DJ AND FREEDMAN LS. (1986). A predictive approach to selecting the size of a clinical trial, based upon subjective clinical opinion. *Stat. Med.*, **5**, 1–13.

SPIEGELHALTER DJ, FREEDMAN LS AND PARMAR MKB. (1993). Applying Bayesian ideas in drug development and clinical trials. *Stat. Med.*, **12**, 1501–1511.

STENNING S AND ALTMAN DG. (1994). Equivalence trials. *Br. J. Cancer* (in press).

WHITEHEAD J. (1992). The design and analysis of sequential clinical trials, 2nd edn. Ellis Horwood: Chichester.

WITTES J AND BRITTAIN E. (1990). The role of internal pilot studies in increasing the efficiency of clinical trials. *Stat. Med.*, **9**, 65–72.

YUSUF S, COLLINS R AND PETO R. (1984). Why do we need some large and simple randomised trials? *Stat. Med.*, **3**, 409–420.